# 

**Published:** 1899-09-02

**Authors:** 


					THE HOSPITAL. " The standard of highest purity."?Lancet. Ukfi'kmbkk 15397
CADBURY'S V K* CADBURY'S
COOOA )%> Jig*? COCOA ,
ABSOLUTELY PURE. NO DRUGS OR ALKALIES USED. C/<-
? BSvm^ i > A
j v :-
4Igf ZQl G F. N Lr R A L'S .
!Klf3lS&P^1 ~i?Q
m inS' JtskSP^ '.;-? ^ ? L ?J O
^^^r^SfULASCOfY WESTERN INFIRMARY
No. 67b. Vol. XXVI. Eajssia
Saturday, Sept. 2nd, 1899.
ivunruLn Ann nunrr/LH HOSPITAL
[s?b ?Pp?soTe,?"] PRICE TWOPENCE.

				

